# Single Molecule Fluorescence Detection and Tracking in Mammalian Cells: The State-of-the-Art and Future Perspectives

**DOI:** 10.3390/ijms131114742

**Published:** 2012-11-13

**Authors:** Marisa L. Martin-Fernandez, David T. Clarke

**Affiliations:** Central Laser Facility, Science & Technology Facilities Council, Research Complex at Harwell, Rutherford Appleton Laboratory, Harwell Oxford, Didcot OX11 0QX, UK; E-Mail: dave.clarke@stfc.ac.uk

**Keywords:** single molecule tracking, mammalian cells, experimental methods, fluorescent labels, feature detection

## Abstract

Insights from single-molecule tracking in mammalian cells have the potential to greatly contribute to our understanding of the dynamic behavior of many protein families and networks which are key therapeutic targets of the pharmaceutical industry. This is particularly so at the plasma membrane, where the method has begun to elucidate the mechanisms governing the molecular interactions that underpin many fundamental processes within the cell, including signal transduction, receptor recognition, cell-cell adhesion, *etc*. However, despite much progress, single-molecule tracking faces challenges in mammalian samples that hinder its general application in the biomedical sciences. Much work has recently focused on improving the methods for fluorescent tagging of target molecules, detection and localization of tagged molecules, which appear as diffraction-limited spots in charge-coupled device (CCD) images, and objectively establishing the correspondence between moving particles in a sequence of image frames to follow their diffusive behavior. In this review we outline the state-of-the-art in the field and discuss the advantages and limitations of the methods available in the context of specific applications, aiming at helping researchers unfamiliar with single molecules methods to plan out their experiments.

## 1. Introduction

Single molecule tracking provides a direct way to quantify dynamic biological events at the single-molecule level without being obscured by the averaging inherent in ensemble experiments. The method can be a powerful method to study, with high spatial and temporal resolution, the dynamic and mechanistic features of molecular interactions that are important for biological function.

Single-molecule imaging was first applied to investigations of molecular interactions involving purified biomolecules immobilized on glass or in artificial lipid bilayers, where sample backgrounds are inherently very low and the density of molecules per unit area easy to control. Among the breakthroughs that followed one can cite, for example, the direct observation of kinesin stepping by optical trapping interferometry [1], or recycling in stationary membrane tubes [2], the measurement of the power stroke size of Myosin V on actin with nanometer accuracy [3], the revelation of a DNA-scrunching mechanism during initial transcription [4], and diffusional migration in single stranded DNA-binding proteins [5]. For more information, see for example [6].

Single-molecule techniques were soon extended to imaging in bacterial cells, e.g., *Escherichia coli*, where controlling gene expression, and therefore the number of proteins per cell can be readily achieved [7]. Cell autofluorescence can also be easily minimized, for example, by growing cells in low-background culture media [8]. These advantages, together with the fact that the entire cell volume (2 μm long and 0.5 μm in diameter [9]) can be within the depth of focus of a standard high numerical aperture (NA) objective (depth of focus 300–700 nm), meant that single molecule detection could be accomplished using standard widefield epifluorescence microscopy. Examples of breakthroughs include the direct observation of steps in rotation of the bacterial flagellar motor [10], determining the stoichiometry and turnover in single, functioning membrane protein complexes [11], the demonstration of translational bursts from a repressed promoter [12], the measurement of the search time for a transcription factor to reach its target site in a living bacterial cell [13], and the stoichiometry and architecture of active DNA replication machinery [14]. For a useful review see [15].

Mammalian cells are much larger than bacterial cells, typically >10 μm × 10 μm long and several μm in depth. They also have significant levels of background autofluorescence, mostly due to the intrinsic fluorescence of molecules like NADPH and flavins [16]. Because mammalian cell depth is much larger than the depth of focus of standard high NA objectives, if imaged using epifluorescence illumination they will produce significant amounts of out of focus autofluorescence background that will swamp the emission from any single molecule of interest. The use of total internal reflection fluorescence (TIRF) [17] (see Section 2.4) together with the availability of brighter and more photostable fluorescence probes, new sample preparation and labeling methods and improved algorithms for feature detection and tracking have allowed single molecule tracking to become in the last decade an important tool to obtain direct information on how molecules diffuse and interact with each other in the mammalian cellular context. This has been particularly so at the plasma membrane, a heterogeneous entity with different structures at the nano and meso (<200 nm) scales [18]. Here, not only has single-molecule tracking allowed unprecedented observations of membrane protein interactions at the molecular level, but it has also significantly contributed to the recent plasma membrane paradigm shift from inert continuum fluid to a partitioned dynamic assembly that plays a key role in mediating protein interactions [19].

Because the fluorescence emission of single molecules is very weak, their detection is critically dependent upon the ability to minimize unwanted background signals. In addition, the diffraction-limited resolution of optical microscopy requires molecular densities of ~less than 1 molecule per μm^2^ so that the ~250 nm diameter image spots from single molecules do not overlap. This means that, for single molecule imaging in mammalian cells, considerable care is required in the selection of cell lines, fluorescence labeling, experimental design, and data analysis. In this review, we summarize the experimental choices facing biologists unfamiliar with single molecule experiments in a mammalian cell system. We also outline the type of information content that can be extracted from the data and give a few examples where these choices were paramount for the success of the experiment.

## 2. Experimental Design

### 2.1. Choosing a Cell Line

**Figure 1:**
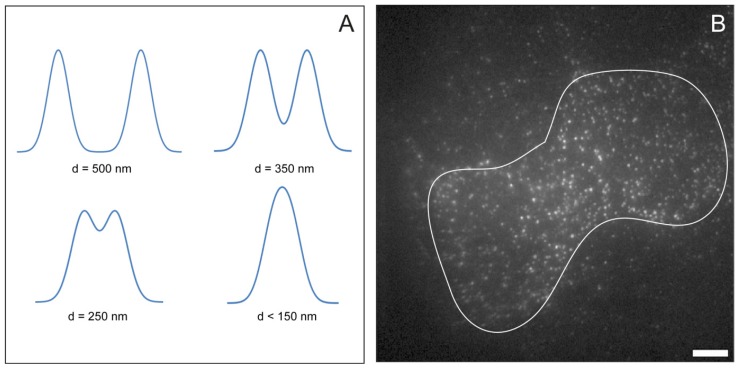
(**A**) Simulated intensity cross sections through a pair of single molecule fluorescence emitters with varying separations. Individual features can only be resolved when the molecules are separated by a distance greater than the point spread function of the microscope; (**B**) Single molecule TIRF image of EGFR in the plasma membrane of HeLa cells (expressing approximately 50,000 EGFR molecules per cell). EGFR are labelled with their ligand EGF, conjugated with the fluorophore Atto 647N (bar 8 μm). The white line shows the approximate cell borders.

Single molecule imaging is ideally suited to investigations in cells expressing low physiological numbers of the protein of interest because this avoids fluorescence from multiple molecules merging and preventing single molecule detection. Molecules must be separated by a distance greater than the point spread function (PSF) of the microscope if they are to be individually resolved. Figure 1A illustrates this effect. Two or more fluorescence emitters located within the PSF will not be detected as individual molecules. The experimenter should aim for molecule densities where clearly separated fluorescent spots are visible, with a significant degree of dark space between them. An example single molecule image from a sample with a good density of fluorescent molecules is shown in Figure 1B. Although the choice of cell model ultimately depends on the biological question(s), as a general guide one typically would like to find a cell line that expresses the protein of interest at a low number of copies per cell. As a rule of thumb, 20,000 protein copies per cell or fewer typically allows the labeling of a significant number of the protein copies at the plasma membrane without their image spots overlapping. [20,21]. It is, however, possible to apply single molecule imaging to cells that overexpress the protein(s) of interest. For example, the epithelial carcinoma A431 cell line overexpresses HER1 (aka epidermal growth factor receptor (EGFR)/ErbB1)), a tyrosine kinase receptor implicated in the onset and development of unregulated cell growth [22], to a level of ~2–3 × 10^6^ copies per cell [23], of which 30% are located at the cell surface [24]. This cell line was the first employed for single molecule studies in mammalian cells [25]. The compromise that has to be made is that only a small fraction of surface receptors can be labeled with fluorophore. This can be a limitation to investigate protein interactions because the lower the fraction of fluorescent proteins the lower the chances of detecting colocalization events. It has also been reported that overexpression can alter protein dynamics at the cell surface [26], so a parallel control on a lower expression system may be required to validate the results. An alternative elegant way to derive single-molecule trajectories in cells overexpressing the protein of interest is to combine photoactivated localization microscopy (PALM) with live-cell single-particle tracking, which uses switchable probes allowing a small fraction of probes to be phototactivated at any one time [27].

On some occasions it may be eminently possible to find a cell line with the desired level of expression of the protein of interest. For example, in the case of HER1-4, a receptor family implicated in the development and progression of most solid tumors, including non-small cell lung cancer, head and neck, breast, colon [28], a significant number of wild-type and stably transfected inmortalised cell lines with various expression levels are available (see for example [29–31]. If finding a cell line with required numbers of protein copies per cell is not possible, then the relevant plasmid DNA has to be transfected into an appropriate cell model. Popular non-viral transient transfection strategies include the use of liposomal based (e.g., Lipofectamine (Life Technologies, Grand Island, NE, USA) and non-liposomal reagents (e.g., FuGENE^®^ HD (Promega, Madison, WI, USA). Optimizing of transfection efficiency for single molecule imaging may, however, be nontrivial. The strength of the promoter is an important consideration because most promoters are optimized for high protein yields. Other parameters to consider are the ratio of transfection reagent to DNA, the amount of transfected nucleic acid, and the length of time cells are exposed to the transfection reagent. Imaging cells only a few hours after transfection, instead of waiting the normal >12–24 h for protein to be expressed, can give good results [32]; however, care should be taken to ensure the cells have recovered from exposure to the transfection reagents, which are toxic (see for example [33,34]). Other options include the use of a viral transfection system which, because of their high levels of gene transfer, compared to non-viral vectors, can result in a much larger fraction of cells labeled at the desired low level (see for example [35]), and the use of DNA transposons, which are a mobile genetic element that efficiently transposes between vectors and chromosomes via a “cut and paste” mechanism (see for example [36]). For long term experiments the best option could be to stably transfect cells with a vector encoding the protein of interest expressed from an inducible promoter; the level of protein expression can then be controlled using different concentrations of an antibiotic (see for example [37]).

### 2.2. Fluorescent Probes for Single Molecule Tracking

Because native proteins are invisible under the optical microscope a probe must be attached for fluorescence detection. Probes of interest include organic molecular dyes, fluorescent proteins (FPs) and quantum dots (QDs), which are semiconductor nanocrystals. The choice of fluorescent probe is guided by a number of general considerations; good probes must have high extinction coefficients, high quantum yields, and good photostability [38]. In multicolour applications narrow excitation and emission spectra are also an advantage (Table 1).

Among the organic dyes, several groups of probes have been used in single molecule tracking experiments, including xanthenes (e.g., Rhodamine), cyanines (e.g., Cy3 and Cy5), and other groups better known by their trademark names, like the Alexa (Life Technologies, Grand Island, NE, USA) and Atto (Atto-Tec, Siegen, Germany) dye series. Because of the inherent background fluorescence of mammalian cells, most studies have used probes that absorb light at wavelengths longer than 450 nm. Currently, the palette of most available probes extends to excitations up to the far red (*i.e.*, <660 nm) (Table 1) although near infrared dyes have also successfully been employed [39]. Advantages of organic dyes are: (i) their small size (typically <1 kDa), which reduces the chances of sterically hindering protein-protein interactions; (ii) their wide commercial availability; (iii) their wide availability in many colors (see for example [40]), although care must be taken to choose dyes whose emission spectra do not overlap to minimize bleedthrough between channels in multicolour tracking applications; (iv) different dyes can display different degrees of sensitivity to the environment (e.g., ionic concentration, pH, viscosity *etc.*) (see for example [41,42]), which can be useful to interrogate some sample properties; (v) they can be covalently linked to proteins using commercially available kits; and (vi) they have dipolar moments that make them suitable for polarization measurements (see for example [43,44]). A limitation of organic dyes is their tendency to stick to the cell substrate; this can be a big challenge in single molecule tracking, where the aim is to derive the diffusion rates of different protein species, because non-specifically bound probes are immobile and can contaminate results [45,46]. In practice, the suitability of a dye will strongly depend on the reagents, sample preparation procedures and the aims of the particular application.

Fluorescent proteins (FPs) are genetically encoded fluorescence markers and can be monomeric (25–30 kDa in size) or multimeric, the latter being proportionally brighter [47]. Best known is the prototypical green fluorescence protein (GFP), in which a fluorescent chromophore 4-(*p*-hydroxybenzyliden)-5 imidazoline forms within a rigid beta barrel during the maturation of the protein in the cell from a tripeptide (Ser65-Try66-Gly67) [48]. A big advantage of FPs is that they can be attached to most proteins using standard molecular biology methods [49]. Most commonly, plasmid vectors are used, introduced into the host cells using lipid vesicles, a process known as lipofection [50]. Details of plasmid design are beyond the scope of this review, but more information can be found in [51], for example. Transfections are either transient, in which expression of the fluorescent gene fusion product occurs several hours after transfection, continuing for 72 to 96 h after introduction of the DNA, or stable, where the cells continue to produce the fluorescent protein indefinitely. Stable cell lines can be selected using antibiotic markers introduced into the plasmid [52]. Creation of stable cell lines is usually achieved by using more efficient techniques at the initial transfection stage, such as electroporation, a method that uses high voltage pulses to introduce pores into the plasma membrane [53]. Probably the most important aspect to consider when attempting to use fluorescent proteins for single molecule work is to keep the expression level low enough to enable individual molecules to be distinguished.

To investigate protein-protein interactions it is advantageous to choose FP versions that do not oligomerise in order to prevent the FP itself being the driver of the dimerisation of the protein of interest, although in practice this means compromising the brightness of probes that are already not too bright. If the resulting SNR is too low, one option to ameliorate the loss in signal is to fuse tandems of monomeric FPs to the protein of interest [54]. In recent years, the color palette has also been enriched through mutations in GFP, creating a number of orange, red and far-red FPs, like for example mOrange2, tagRFP, mKate2, *etc.*, which have not only higher extinction coefficients, but also more pH-stability, higher brightness and photostability (for a review see [55]). There are also several photoactivatable and photoswitchable FPs available, e.g., Dendra2, mEos2, *etc.*, ideal for super-resolution methods (see for example [56]).

One key limitation shared by organic dyes and FPs is their poor resistance to photobleaching ([38] and references therein). The most photostable among them typically undergo about 10^5^–10^6^ excitation-deexcitation events prior to bleaching, which means they emit a maximum of 10^5^–10^6^ photons. The finite number of photons available implies that single-molecule fluorescence measurements are generally limited by shot noise [57]. This means that the signal-to-noise ratio (SNR) is equal to √*N*, where *N* is the number of photons recorded in a frame of data. A number of factors contribute to achieving the highest SNR, and these need to be optimized for the particular type of experiment being performed. Important factors include the excitation intensity, the length of the frames, the efficiency of the detector, the total number of frames and the lag time between them, and the photophysical characteristics of the dye or fluorescent protein. For example, increasing the photon emission rate via stronger illumination will allow faster frame rates at a high SNR but only at the expense of speeding up the eventual bleach. Of course, frame rate selection is also dependent on the behavior of the features being tracked, so, for example, more rapidly diffusing molecules require faster tracking and therefore higher frame rates. Depending on the duration of the phenomena under investigation, the optimal illumination power must be carefully chosen to balance out SNR, frame rate and bleaching times. Using illumination powers of <2 mW, the best organic dyes can last up to a few minutes and the best FPs up to a few tens of seconds (see for example [44,58]). Another key limitation is the susceptibility to blinking, defined as intercalated periods where the fluorescence intensity reversibly drops to zero [59]. As with photobleaching, blinking can often be related to the presence of a long-lived dark triplet state. Blinking is undesirable in single molecule tracking as features cannot be detected for the duration of the off periods. Given that the methods used to palliate blinking, like the introduction of redox cocktails [60,61], are not generally applicable to the physiological-like conditions of live cell work, the photophysical properties of the tag are key criteria when choosing an organic dye or FP for single molecule tracking experiments. (It should be noted that the presence of significant blinking is in contrast an advantage when employed in other super-resolution imaging techniques such as PALM and STORM [62–64]).

Because of their exceptional resistance to photobleaching and their very high extinction coefficient and brightness, QDs have been widely used in biology [65,66]. QDs provide big advantages in single molecule tracking when the inherent SNR is poor (for example to follow intracellular processes where TIRF illumination cannot be used [67]) and when molecular behavior needs to be monitored for extended periods (see for example [68,69]). QDs also have a broader excitation spectrum [70] which can be both an advantage if one wants to simultaneously excite different probes with the same laser source, and a limitation if multiplexing is required. Their narrower, size-tunable emission spectra are ideal for applications in which many colors are required, for example to distinguish several protein species. Limitations of QDs include their relatively large size (~20 nm), which may interfere with some protein-protein interactions, that they display significant blinking, and that it is difficult to label the protein of interest at a 1:1 stoichiometric ratio [71], although in some systems this could be an advantage [72]. An elegant method of conjugating QDs to surface proteins in living cells is via the use of *E. coli* biotin ligase [73].

### 2.3. Labeling the Proteins of Interest

If fluorescent proteins cannot be used to label the proteins of interest, other ways must be used to attach fluorescent molecules. Some integral plasma membrane proteins (e.g., transmembrane receptor tyrosine kinases, G-protein coupled receptors, cytokine receptors, ion channels, *etc*.) can be labeled at their extracellular domains by adding to the culture medium a fluorescent derivative of a high-affinity protein ligand that can avidly recognize a specific site in the receptor with a known stoichiometry. The ligand carrying the fluorescent probe can be, among others, a receptor agonist (e.g., a growth factor ligand, (e.g., [25]), a cytokine (e.g., [74], *etc*.), an antagonist (e.g., an affibody [75], a small drug compound or antibiotic [20]), or a genetically engineered antibody mimetic protein (e.g., DARP in [76]). If the objective of the experiment is to determine the stoichiometry of a complex, or measure the number of molecules in a cluster, it is important that the ligand is labeled in a specific residue at a 1:1 stoichiometry. In this way, one avoids steric variations from one labeled ligand to another. On the other hand, for tracking experiments it may be preferable to increase the labeling ratio so that longer tracks can be recorded before the label is completely photobleached.

**Figure 2:**
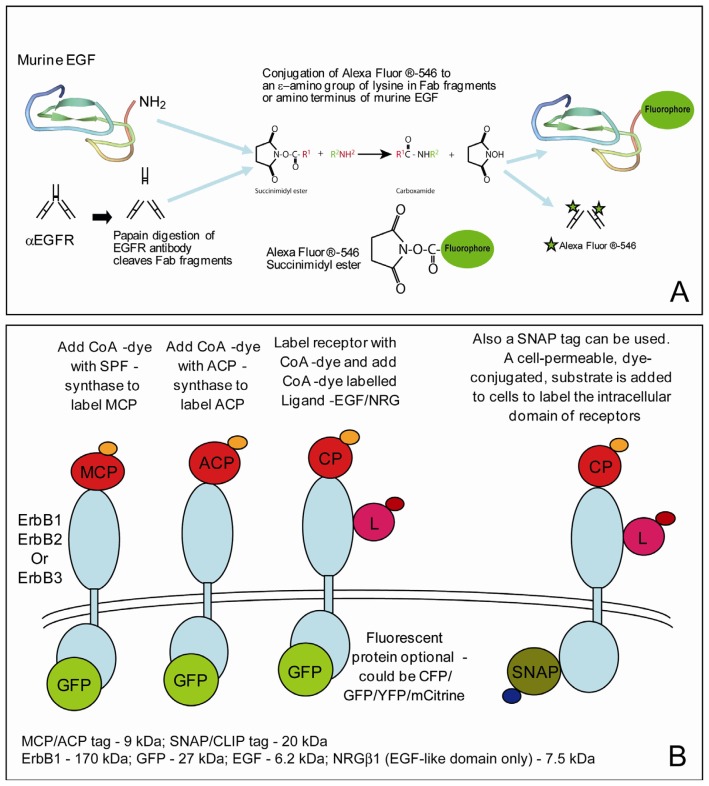
Illustration of methods used for fluorescence labelling. (**A**) Conjugation of fluorophores to ligands and antibodies; (**B**) The use of combined methods for multiple labelling of proteins with different ligands: The intracellular domain is labelled with green fluorescent protein and/or SNAP tags, while the extracellular domain is labelled using a combination of MCP, ACP, and CLIP tags, and chemically-conjugated ligand.

Ligands can be chemically labeled using, for example, succinimidyl ester derivatives of fluorescent dyes that provide an efficient and convenient way to covalently cross-link the fluorophore specifically to primary amines (R–NH_2_) in the ligand, found in lysine residues and at the *N*-terminus. Similarly, maleimide derivatives can be used to label thiol groups (R–S–H), present in cysteine residues (Figure 2A). The desired 1:1 dye/ligand stoichiometry is easy to achieve if ligands do not have any lysine residues, such as, for example, the epidermal growth factor (EGF) ligand which binds the extracellular domain of HER1, because the dye can only bind the *N*-terminus. Similarly, 1:1 stoichiometry can be easily obtained with ligands that have a single cysteine residue, such as, for example, affibody ligands. If two cysteines are present, it is often possible to mutate one to another small amino acid whilst maintaining good affinity for the receptor.

If the proteins cannot be labeled by adding fluorescent probes to the culture medium one requires molecular biology methods to engineer protein-tag fusions. Protein tagging techniques involve the addition of a peptide sequence to the protein of interest. The peptide sequence is designed to be susceptible to a specific chemical reaction, in the case of fluorescence measurements enabling the attachment of a specific fluorophore. The SNAP tag is a 20 kDa mutant of the DNA repair protein *O*6-alkylguanine-DNA alkyltransferase [77]. It reacts specifically with derivatives of benzylguanine, which can be used to covalently attach a fluorescent probe. Similar to the SNAP tag is the CLIP tag. This is a version of the SNAP tag that has been modified to react with a different substrate, *O*2-benzylcytocystine [78]. By using combinations of CLIP and SNAP tags, it is possible to label a single fusion protein with two different fluorescent probes. Many of the substrates of CLIP and SNAP tags are membrane-permeable, so they can be used for labeling of intracellular as well as extracellular proteins.

A slightly different approach is taken with another two tags, ACP and MCP. In this case, the protein is fused to an acyl carrier protein. The fusion protein can then be labeled with derivatives of Coenzyme A (CoA), via a post-translational modification catalysed by the enzyme AcpS. An advantage of the ACP tag is its relatively small size (9 kDa) [79]. A mutant of ACP, known as MCP, is modified by the enzyme Sfp, but not by AcpS, allowing, in a similar way to SNAP and CLIP tags, the labeling of a single fusion protein with two fluorophores. Unlike SNAP and CLIP, the substrates of ACP and MCP tags are not membrane permeable, so are only suitable for labeling proteins on the surface of the cell. The principles of labeling with protein-tag fusions are illustrated in Figure 2B.

Although the tags described above are smaller than fluorescent proteins, they are still relatively large and have the potential to interfere sterically with protein function. Other tags, known as FlAsH (green) and ReAsH are smaller, but have relatively poor labelling specificity and can be toxic to cells [80]. Recently, other methods have been demonstrated for the specific labelling of proteins within cells. Probe Incorporation Mediated by Enzymes (PRIME) uses a mutant “fluorophore ligase” that is able to attach the blue fluorophore 7-hydroxycoumarin to proteins fused to a specific recognition sequence [81]. Similarly, a two-step method has been described that uses *E. coli* lipoic acid ligase to site-specifically ligate a trans-cyclooctene derivative onto the protein of interest, followed by derivitization with a tetrazine-fluorophore conjugate [82]. These methods have the potential to overcome some of the difficulties associated with conventional tag-labelling, but have not so far been applied in single molecule studies.

### 2.4. Optical Set up

**Figure 3:**
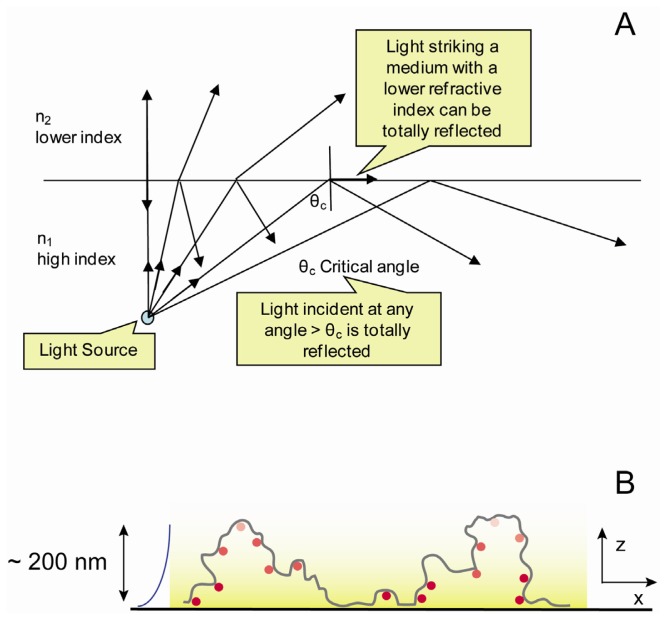
(**A**) Illustration of the principle of Total Internal Reflection Fluorescence (TIRF) microscopy. (**B**) Effect of TIRF illumination on fluorescent molecules in the plasma membrane of adherent cells; molecules close to the cover slip receive more intense illumination, and there is no illumination beyond a depth of approximately 200 nm.

One of the major challenges in imaging single molecules in mammalian cells is obtaining data with sufficiently high SNR in the presence of high levels of background fluorescence. To detect single molecules cell autofluorescence must therefore be minimized. In mammalian cells, this is almost always accomplished by the use of total-internal-reflection fluorescence (TIRF) [83] (Figure 3A). Because of the difference in refractive index between the glass substrate and the cell culture medium, light that hits the glass-water interface at or beyond the so-called critical incidence angle cannot propagate towards the sample and is totally internally reflected. TIRF illumination creates an evanescent excitation field on the glass coverslip to which the cells adhere which reduces exponentially with depth, penetrating ~200 nm axially into the sample. The evanescent field differentially excites molecules in the vicinity of the cell surface reducing intracellular autofluorescence background to a level where single-molecule detection becomes possible [84]. In the case of mammalian cells this means that fluorescence excitation is restricted to membrane proteins and proteins in the close vicinity of the plasma membrane, with minimal fluorescence from molecules deeper inside the cell (Figure 3B).

TIRF illumination can be achieved in one of two ways. Prism-based TIRF microscopy uses a prism attached to the coverslip to direct a light beam towards the glass-water interface at the critical angle [17]. In objective-based TIRF microscopy, an objective with very high numerical aperture (NA > 1.45) is used to achieve illumination at the critical angle, by placing the laser illumination off the centre of the objective’s back focal plane [85]. The latter method is now more commonly employed because of convenience of sample handling and ease of adjustment of the incident angle.

**Figure 4:**
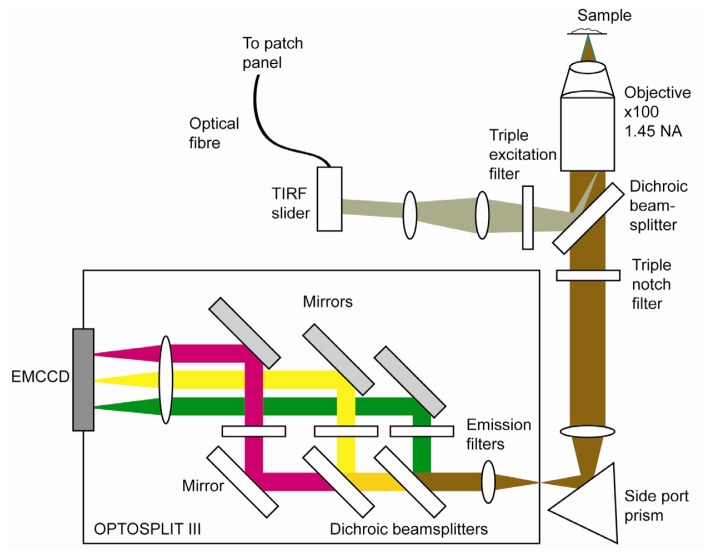
Schematic view of a microscope set up for 3-colour single molecule TIRF microscopy.

The layout of a typical objective-based TIRF single-molecule microscope is shown in Figure 4 (see for example [86]). This microscope is designed for excitation of fluorescence at three wavelengths, and its detection in three channels. A typical use of a system of this type might be the investigation of interactions between three different protein species diffusing on the surface of a cell. Lasers of different colors can be delivered to the sample in a number of ways, including via the use of a polarisation maintaining wavelength multiplexer, a laser engine, or a supercontinuum laser source [87]. The combined laser output is launched into a polarisation maintaining single mode fiber, which is plugged into an inverted microscope through a polarisation dependent TIRF slider that allows both TIRF and epifluorescence illumination. Maintaining the polarization of the laser(s) not only reduces losses at the TIRF slider but also allows delivery of polarization components along the *z* and *x* axis with respect to the sample that can be used to derive protein orientation information [44]. TIRF attachments are available from all major microscope manufacturers, although it is also possible to custom-build optics for TIRF illumination (see for example [88]). The laser beam is reflected into the high NA objective by suitable triple band filter sets. Fluorescence from the sample is imaged onto the entrance aperture of an Optosplit III image splitter (Cairn Research). This divides the image into three spectrally distinct but spatially identical components, which are imaged side by side on an electron multiplication CCD (EMCCD) camera. To date, EMCCDs are the most suitable detectors for single molecule imaging because of their combination of high signal-to-noise with relatively fast readout speeds [89].

Finally, it should be noted that the increased molecular contrast provided by the limited depth of TIRF illumination could also be considered as a limitation if there is a need to image molecules that are located in the cell cytoplasm or nucleus, not close enough to the plasma membrane to fall within the TIRF illumination field. Besides using QDs [67], a way forward for intracellular single molecule imaging might be to use fluorescent probes which are excited by infrared radiation, at wavelengths where little cell autofluorescence occurs. A recent publication has showed the use of conventional epifluorescence illumination to image single molecules labeled with infrared probes, inside cells [39].

### 2.5. Feature Detection, Localisation and Analysis

Data analysis is a key part of any single molecule experiment. It is beyond the scope of this review to cover all the aspects of single molecule data analysis, but in this section we briefly discuss some of the more important points for single molecule detection, localization, and tracking. We refer to a number of papers in which the reader may find more detailed information if required.

Significant effort has been applied in the last few years to the development of data analysis methods suitable for single molecule tracking studies in cells. The general approach, outlined below, can be divided into two steps: (i) the detection and measurement of features in each image frame; and (ii) the inferring of information on individual molecular movement (tracks) from the changes in these features through time. Key challenges include the presence of crowded fields, fluorophore blinking, low SNRs and poor signal-to-background ratios, the latter because signals from single molecules are superimposed on out-of-focus fluorescence from other molecules and residual cell autofluorescence not completely eliminated by TIRF illumination. In addition, because fluorophores located at varied axial distances in the cell membrane experience different excitation field strengths, a side effect of the limited depth of the evanescent field is that the SNR ratio varies from molecule to molecule.

To detect single molecules these have to be distinguished from the surrounding background. This is often a non-trivial procedure as the background may be neither flat nor smooth, and may show features that resemble single molecules. Single molecules can be considered single point emitters because their size is much smaller than the optical wavelengths employed. Feature detection relies on the knowledge that the diffraction-limited spot image of a single molecule is equal to the point spread function (PSF) of the microscope. Features are therefore only considered to arise from a single molecule if they have a size equal to the PSF of the microscope. Even though the true theoretical profile of the PSF is actually an Airy disc, a Gaussian profile with fixed known width has normally been adopted as an approximation for the PSF of the microscope (see for example [90–93]). This is because a Gaussian is significantly easier to use and computationally faster to evaluate, while the results are often indistinguishable. Deconvolution of the Gaussian profile allows determination of the position of the single molecule emitter with precision of up to 1–2 nm depending on SNR and the amount of background present [3]. (It should be noted that, in combination with the availability of photoactivatable probes, this is the principle behind the development of super-resolution microscopy; for a recent review see for example [94]).

If the SNR and signal-to-background ratio are reasonably good, popular single molecule feature detectors use combinations of thresholding methods and step detection (single molecules photobleach in one step and this allows counting of the molecules in a PSF and distinguishing them from the background) (e.g., [18,20,21,32]). At low SNRs, tracking algorithms that perform best incorporate likelihood-based feature detection algorithms [95] or Bayesian segmentation [96], which objectively compare the probabilities of a feature being present with the null hypothesis of just background. One key remaining challenge of feature detection is how to satisfactorily address the high particle density typical of mammalian cell images, which is often present even in cells that express low numbers of protein copies per cell when a substantial fraction of these proteins are labeled [20,21]. Towards this goal, a feature detector from astronomy was recently shown to perform well when applied to crowded conditions [97].

Tracking is the process of following molecules through time to derive the time course of their intensity and position. This enables single molecule data to be analyzed in live cells to derive information on the characteristics of the molecular movement over many frames and measure diffusion rates for individual molecules. When performed in two of more color channels, tracking can also determine the presence of molecular interactions and their kinetic rates. The latter requires the use of multicolor feature detection and tracking, which typically requires the application of highly precise channel registration procedures (e.g., [98]). Exploiting the temporal information from the data is often achieved by tracking detected features from frame to frame and interpreting the resulting position and intensity traces *versus* time. In some cases though, information on protein diffusive behavior can be derived by measuring correlation properties without first calculating temporal tracks [99] or by separately considering the cases of moving and non-moving proteins and performing simple tracking [90]. The correlation method is computationally cheap, robust, and can be applied to high particle densities, without the requirement for a priori knowledge of the dynamic coefficients.

One key challenge in tracking is that the particles under observation exhibit temporary disappearance, e.g., from blinking, detection failure, merging and splitting events from dimer-monomer transition, or because the separation between features goes below and then above the diffraction limit. As a result, the feature detector may not detect features in all frames, leading to tracks with ‘gaps’ from which the “true” tracks have to be recovered. Given that the global spatio-temporal solution that would address this challenge is computationally prohibitive, most feature trackers use a number of heuristic algorithms to derive a computationally tractable global spatial solution. However, this approach tends to optimize for longest track length as it is impossible to determine the relative likelihood of a set of short tracks rather than one tracked formed by linking them [100]. This problem was statistically addressed by Sergé *et al*. [101], who looked at the probability of tracks forming a set of tracks with maximum likelihood. Jaqaman *et al*. [95] proposed a maximum likelihood method of parameter selection and developed a set of heuristics for determining these models and joining the tracks together. Despite the progress made, a computationally tractable, global spatio-temporal solution still remains to be found.

## 3. Information Content

Single molecule tracking is uniquely suited to investigate three molecular properties: First, from the quantum photobleaching of the fluorescence tag attached to the protein it can report on stoichiometry [11,102,103]; second, from the position of interacting molecules *versus* time it can identify the presence or absence of an interaction [20,21,69]; third, from the different modes of molecular diffusion it can provide information on the interaction of a molecule with its surroundings. Given that the latter property is the least intuitive of the three, we briefly introduce below some of the key concepts.

The original model proposed to describe the diffusion of proteins in a lipid membrane is the Saffman-Delbrück theory [104]. This model describes a lipid membrane as a thin layer of viscous fluid, surrounded by a less viscous bulk liquid and predicts that molecular diffusion coefficients depend on the radius and height of the diffusing object and the viscosity of the membrane and its surrounding fluid. According to this model, the diffusion of membrane proteins would behave as stochastic molecular transport of the type that can be explained by Brownian motion. The prediction is consistent with diffusive processes at the plasma membrane being ergodic, *i.e.*, one in which the statistical properties of the diffusion behavior (such as its mean and variance) should not be time dependent [105].

**Figure 5:**
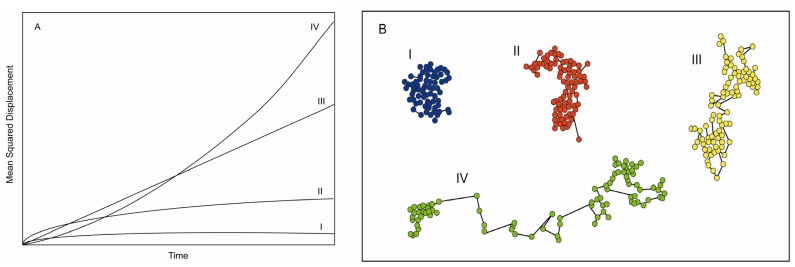
(**A**) Simulated mean squared displacement plots for particle tracking. I shows diffusion for confined molecules, II shows obstructed diffusion, III normal diffusion, and IV shows MSD for directed motion; (**B**) shows example particle tracks for the motion types plotted in (**A**) [115].

Both single molecule and ensemble-averaged methods, like fluorescence recovery after photobleaching (FRAP) [106], have reported the diffusive properties of proteins. These experiments have demonstrated that diffusion is in fact non-ergodic and have shown the presence of anomalous diffusion not explained by the Saffman-Delbrück model [107]. Assuming that movement is random, and approximating the plasma membrane to a 2-D surface, one can derive the molecular mean square displacement (MSD) from the ensemble average diffusion coefficient (*D*). A Brownian particle in this space should display a motion described by a linear MSD, *i.e.*, MSD ∞ *Dt*, where *t* is the time during which molecules are observed). Motions other than normal diffusion will result in non-linear MSD plots (Figure 5). At the plasma membrane is often found that the power law governing MSD motion is sublinear, *i.e.*, MSD ∞ *Dt*^γ^, where γ < 1. This diffusive behavior is referred to as confined motion, or anomalous subdiffusion. In some cases, it is also found that γ > 1, which is referred to as anomalous superdiffusion. Experimental and theoretical work suggests that possible reasons behind anomalous subdiffusion include obstruction due to macromolecular crowding, compartmentalization and fence-like structures at the plasma membrane [108–110], cholesterol-dependent membrane heterogeneity [111,112], and protein binding to the cell cytoskeleton (see for example [69,72,113]). Anomalous superdiffusion arises when the fluorescent molecule is being actively transported, e.g., on microtubules [114]. This gives rise to MSD plots similar to that shown in Figure 5A.

The insights provided by single molecule tracking go beyond those derived from ensemble methods by allowing the investigation of transient events and positional inhomogeneity, which may ultimately explain the mechanism(s) behind hindered diffusion. From single molecule tracks the MSD can be derived for individual molecules from the sum of the square of the molecular displacements (jumps) per frame normalized by the total number of jumps. MSD can be calculated using the formula:

(1) $$MSD(\Delta t)=\langle |r_{i}(t+\Delta t)-r_{i}(t){|}^{2}\rangle$$

where |*r_i_* (*t*+Δ*t*)− *r_i_* (*t*)| is the distance traveled by molecule *i* between time *t* and time *t* + Δ*t*, and the expectation value is over all pairs of time points separated by Δ*t* in each molecular track.

In cases where individual traces are too short for meaningful MSDs to be calculated, the MSD can instead be determined by pooling all the traces together. MSD data from single molecule experiments are already beginning to test the sizeable theoretical framework that has already being laid down to quantitatively explain the effects of lateral diffusion in the presence of immobile obstacles, finite and infinite hierarchy of traps (representing, for example, protein binding sites at the membrane), percolation thresholds, *etc.* (see for example [116,117]). The theory predicts how subtleties in the behavior of molecules may depend on the organization of its surrounding environment. For example, a suitable infinite hierarchy of traps leads to anomalous diffusion at all times, as the protein can never escape from bouncing between traps; in contrast, a finite hierarchy of traps leads to anomalous diffusion at short times and normal diffusion at long times, for example, a protein crosses the barrier between a lipid raft full of traps and another area in the membrane where traps are not present.

## 4. Examples of Applications

There are many examples of the successful application of single molecule tracking to investigate molecular behavior in the plasma membrane of mammalian cells in culture. To cite just a few, it has reported the binding dynamics of EGFR dimers [118], the stoichiometry of M_1_ muscarinic receptor complexes [20], different structures for high- and low-affinity EGFR [119], the diffusion dynamics of single kinesin molecules moving in microtubules [120], the kinetic rates and equilibrium constant of a chemotractant G-protein couple receptor (GPCR) [21], a retrograde transport of EGFR from filopodia to the cell body [68], the dimerization dynamics of EGFR and the enrichment of this receptor in the cell periphery in an actin and receptor-expression-dependent fashion [72], and how EGFR dimerization is promoted by domain co-confinement and stabilized by ligand-binding [69].

In this section we use two of these examples to illustrate the variety of choices that can be made in the experimental design, data collection and data analysis to answer different biological questions. The first example is a bench mark for sample preparation technique [20]. In this work Hern *et al*. investigate the formation and dissociation of dimers of the M_1_ muscarinic receptor, a GPCR which is a subtype of the muscarinic acetylcholine receptor family [121]. The main biological question was whether M_1_ receptors existed as obliged dimers or higher oligomers. Single molecule tracking can provide this information because two molecules in a dimer will show two bleaching steps and one molecule one bleaching step. The number of traces of each kind can be counted in a histogram to derive the percentages of each species. Key to measure the stoichiometry of this interaction is their use of a high-affinity (35 pM) M_1_ antagonist (telenzepine) bound to one of the most photostable and long lasting organic dyes available, Cy3. The slow dissociation rate of telenzepine allowed long term systematic washing of samples to remove of non-specific probes bound to cells and glass, important to derive the proportion of monomers and dimers (note that non-specific probes will be static and show single step bleaching). Also critical for success was the use of CHO cells stably expressing receptors at low physiological levels (1 pmol receptor per milligram membrane protein) that together with the high-affinity of the probe allowed up to 97% of receptors to be labeled using probe concentrations in the range of 0.1–1 nM. The high fluorescent receptor fraction was crucial to quantify the population of dimers and monomers from molecular photobleaching and track colocalisation events and the low concentrations of probe required were in turn crucial to minimize non-specific binding.

The second example is a benchmark in data analysis [69]. In this work, Low-Nam et al captured the dimerization of EGFR in real time and developed sophisticated analytical methods to extract reaction kinetics and characterize monomer and dimer behavior. The sample preparation consisted of using A431 cells overexpressing EGFR where some receptors were labeled with a QD either via an activating ligand or an antagonist. They investigated receptor dynamics by measuring the colocalization of QDs of two colors. The brightness of the QDs allowed nanometer-like precision in the determination of molecular position within each channel by deconvoluting the Gaussian profile of the PSF in each frame of each track. An accurate channel registration procedure was applied to achieve this. As a result they were able to measure the separation between receptors *versus* time in the nanoscale and used these data to identify dimers from pairs of molecules tracking together where the separation between them was consistent with that expected in dimers from crystallographic data. Interestingly, other associations were also found in which receptors were co-confined at the plasma membrane but at a distance too far apart to be consistent with dimerization. Using drugs that disrupt actin polymerization they were able to identify that the plasma membrane architecture and interactions with actin are involved in co-confinement and promote repeated interactions. The photostability of the QD probes was critical for the latter as they allowed long term observations of the same molecules.

## 5. Conclusions and Future Perspectives

We have illustrated some experimental choices facing researchers using single molecule methods in mammalian cells in culture, summarized the advances made in optimizing methods in the fields of sample preparation, data acquisition and data analysis, and review a number of scientific achievements. Some key challenges still remain. It is necessary to improve transfection methods to make it easier (and quicker) to obtain the cellular expression levels optimal for different single molecule experiments. Improving the photostability and brightness of organic dyes and FPs and increasing the color palette of microscopes, toward hyperspectral imaging, will help to link single molecule methods with systems biology. There is also the need to improve feature detection and tracking to better cope with crowded fields and reduce the reliance on heuristics and a priori knowledge to fill in the voids between tracklets in a data set.

Another challenge is posed by a new range of samples employed in single molecule experiments. Single-molecule visualization has now also been applied to differentiated cells (spermatozoa) [122], primary zebrafish embryonic stem cells [123], salivary gland cell nuclei of *Chironomus tentans* larvae [124], and plant cells in Arabidopsis leafs and roots, where it was hitherto believed that TIRF was not possible (see for example [125]). These developments have extended single molecule investigations to molecular phenomena in conditions as close as possible to physiological, *i.e.*, in the multi-cellular and *in vivo* context, where the cell’s environment includes other cell types and extracellular matrix which affect molecular processes in order to maintain tissue specificity and homeostasis (Kleinman *et al*. 2003). This is an important step because molecular behaviour in cultures cells may not always be reflective of the behaviour *in vivo*. However, these new type of samples bring their unique experimental challenges, for example increased levels of autofluorescence, different requirements for molecular expression and sample labeling and, crucially, the need to get deeper into the sample, which in some cases precludes the use of TIRF methods. The next few years promise very exciting developments in the field.

## Figures and Tables

**Table 1:** Characteristics of fluorescent probes.

	Resistance to photo-bleaching	Brightness	Level of blinking	Emission range (nm)	Suitable for polarization	Narrow fluorescence bands
Quantum dots	√√√	√√√	√	400–800	NO	√ (excit.) √√√ (emiss.)
Organic dyes	√√	√√	√	400–800	YES	√√√
Fluorescent proteins	√	√	√	450–650	YES	√√ (excit.) √√√ (emiss.)
